# Editorial for the Special Issue: “Biomimicry and Functional Materials—First, Second, and Third Editions”

**DOI:** 10.3390/biomimetics9070437

**Published:** 2024-07-17

**Authors:** Tun Naw Sut, Bo Kyeong Yoon

**Affiliations:** 1 Translational Nanobioscience Research Center, School of Chemical Engineering, Sungkyunkwan University, Suwon 16419, Republic of Korea; 2 School of Healthcare and Biomedical Engineering, Chonnam National University, Yeosu 59626, Republic of Korea

## 1. Introduction

Nature has long been a source of inspiration for innovation in materials science. This Special Issue on “Biomimicry and Functional Materials” brings together a collection of research articles that explore how biological systems can inspire the design and development of new materials. We are pleased to highlight a number of diverse studies that span from fundamental research to practical applications and showcase the high-impact potential of this interdisciplinary field.

## 2. First Edition

The first edition of our Special Issue introduced research on biofunctional materials, demonstrating how the unique properties of biological materials can be replicated and enhanced for technological applications. These studies showed that biomimetics research can lead to significant improvements in material performance, including increased strength, flexibility, self-healing capabilities, and biocompatibility. Findings from the first edition are listed below.

- **Decellularization of Pericardia**: A novel detergent was developed to enhance the decellularization of bovine and porcine pericardial tissues, effectively removing cells while preserving the extracellular matrix, which is crucial for heart valve replacements [Contribution 1].
- **Probiotic Encapsulation**: A cellulose–chitosan hybrid encapsulation method significantly improved the viability and stability of probiotics during simulated gastric transit and in kefir, suggesting a promising approach for probiotic delivery in functional foods [Contribution 2].
- **Poly(ε-caprolactone)/Reduced Graphene Oxide Scaffolds**: Research demonstrated that adding graphene oxide to biodegradable poly(ε-caprolactone) scaffolds enhances their mechanical properties and thermal stability, making them suitable for tissue engineering [Contribution 3].
- **Bone Defect Healing**: An in vivo animal study examined the effects of diclofenac and simvastatin on bone defect healing, finding that diclofenac impairs healing while simvastatin promotes bone regeneration, which highlights the importance of drug selection in bone injury treatment [Contribution 4].
- **Dental Implants**: The attachment and proliferation of human gingival fibroblasts on titanium disks were found to be influenced by surface roughness, with moderate roughness leading to better outcomes, which is vital for the success of dental implants [Contribution 5].

## 3. Second Edition

The second edition featured a broad range of applications and theoretical insights into the synthesis and characterization of biomimetic materials and their applications as adhesives, coatings, and sensors, listed as follows.

- **Silk-Based Hydrogels**: Their potential was highlighted for tissue engineering due to their excellent biocompatibility and mechanical properties, making them ideal for regenerative medicine applications [Contribution 6].
- **Gelatin-Based Edible Films**: A comparative study on gelatin-based edible films sourced from porcine and bovine gelatin and infused with spearmint essential oil demonstrated differences in tensile strength and antimicrobial properties, suggesting potential improvements in food packaging solutions [Contribution 7].
- **Extracellular Matrix Extraction**: The process of extracting extracellular matrix components from tilapia bones using hydrochloric acid was optimized through a time-dependent demineralization study, providing valuable insights for biomedical applications [Contribution 8].
- **Cardiovascular Biomaterials**: A novel biomimetic approach was developed to create calcinosis-resistant glutaraldehyde-fixed biomaterials for cardiovascular surgery, potentially enhancing the durability and performance of cardiovascular implants [Contribution 9].
- **Nanocomposite-Based Hydroxyapatite**: An evaluation of nanocomposite-based hydroxyapatite confirmed its effectiveness for biomedical uses, particularly in bone repair and dental applications [Contribution 10].
- **Cholesterol-Enriched Hybrid Lipid Bilayers**: The formation of cholesterol-enriched hybrid lipid bilayers on inverse phosphocholine lipid-functionalized titanium oxide surfaces showed promise for improving the integration and functionality of medical implants [Contribution 11].

## 4. Third Edition

The third edition covered topics such as responsive and adaptive materials that mimic biological responses to external stimuli and sustainable materials inspired by natural processes. These are listed below.

- **Skin Tissue Regeneration and Electronic Skin**: Biomimetic materials have shown promising results for wound healing and wearable electronics [Contribution 12].
- **Structural Coloration of Silk**: A study on the preparation of natural plant polyphenol catechin films demonstrated their effectiveness in enabling the structural coloration of silk fabrics, providing an eco-friendly method for fabric dyeing [Contribution 13].
- **Antimicrobial Lipid Mixtures**: Investigations into how antimicrobial lipid mixtures disrupt virus-mimicking lipid vesicles using the QCM-D technique provided deeper insights into viral disruption mechanisms, informing the development of new antiviral strategies [Contribution 14].
- **Bone Regeneration Scaffolds**: The development of lamellar septa-like structured carbonate apatite scaffolds exhibited layer-by-layer fracture behavior, enhancing bone regeneration by mimicking the natural structure and strength of bone [Contribution 15].
- **Controlled Drug Delivery**: A kinetic model of fluorescein release through bioprinted polylactic acid membranes was established, offering a new approach to controlled drug delivery systems [Contribution 16].
- **Rapid Biomimetic Coating for Bone Regeneration:** A step-wise method using calcium carbonate vaterite as a precursor forms carbonated apatite on surfaces within 4 h, enhancing bioactivity and efficiency in bone regeneration applications [Contribution 17].

## 5. Conclusions

A key theme in this Special Issue is the integration of multidisciplinary approaches to address complex challenges. By combining insights from biology, chemistry, physics, and engineering, researchers are developing materials that mimic and sometimes outperform their natural counterparts. This cross-disciplinary collaboration is essential for advancing the field and creating practical, scalable solutions.

The contributions in these three editions reflect the vibrant and rapidly evolving nature of biomimicry and functional materials research. The innovative studies presented here not only enhance our understanding of bioinspired materials but also pave the way for future discoveries and applications across various industries, including healthcare and environmental management.

